# Barriers in the implementation of isoniazid preventive therapy for people living with HIV in Northern Ethiopia: a mixed quantitative and qualitative study

**DOI:** 10.1186/s12889-016-3525-8

**Published:** 2016-08-19

**Authors:** Gebrehiwot Teklay, Tsigemariam Teklu, Befikadu Legesse, Kiros Tedla, Eveline Klinkenberg

**Affiliations:** 1 Department of Pharmacy, College of Health Sciences, Mekelle University, Mekelle, Ethiopia; 2 Tuberculosis Program, Tigray Regional Health Bureau, Mekelle, Ethiopia; 3 Department of Public Health Sciences, Karolinska Institute, Solna, Stockholm County, Sweden; 4 Institute of Biomedical Sciences, College of Health Science, Mekelle University, Mekelle, Ethiopia; 5 KNCV Tuberculosis Foundation, Addis Ababa, Ethiopia; 6 Department of Global Health, Academic Medical Center, University of Amsterdam, Amsterdam Institute for Global Health and Development, Amsterdam, The Netherlands

**Keywords:** Challenges, Isoniazid stock out, IPT coverage, Tigray

## Abstract

**Background:**

Isoniazid preventive therapy is a key public health intervention for the prevention of tuberculosis disease among people living with HIV. Despite the confirmed efficacy of isoniazid preventive therapy and global recommendations existing for decades, its implementation remains limited. In resource constrained settings, few have investigated why isoniazid preventive therapy is not implemented on full scale. This study was designed to investigate the level of isoniazid preventive therapy implementation and reasons for suboptimal implementation in Tigray region of Ethiopia.

**Methods:**

A review of patient records combined with a qualitative study using in-depth interviews and focus group discussions was conducted in 11 hospitals providing isoniazid preventive therapy in the Tigray Region. The study participants were health providers working in the HIV clinics of the 11 hospitals in the province. Health providers were interviewed about their experience of providing isoniazid preventive therapy and challenges faced during its implementation. All conversations were audio-recorded. Record review of 16,443 HIV patients registered for care in these hospitals between September 2011 and April 2014 was done to determine isoniazid preventive therapy utilization. Data were collected from April to August 2014.

**Results:**

Fifty health providers participated in the study. Overall isoniazid preventive therapy coverage of the region was estimated to be 20 %. Isoniazid stock out, fear of creating isoniazid resistance, problems in patient acceptance, and lack of commitment of health managers to scale up the program were indicated by health providers as the main barriers hindering implementation of isoniazid preventive therapy.

**Conclusion:**

Implementation of isoniazid preventive therapy in Tigray region of Ethiopia had low coverage. Frequent interruption of isoniazid supplies raises the concern of interrupted therapy resulting in creation of isoniazid resistance. Health managers, drug suppliers and partners working in HIV and tuberculosis programs should be committed to ensure an uninterrupted supply of isoniazid and full scale implementation of isoniazid preventive therapy to eligible people living with HIV.

**Electronic supplementary material:**

The online version of this article (doi:10.1186/s12889-016-3525-8) contains supplementary material, which is available to authorized users.

## Background

Tuberculosis (TB) preventative therapy for people living with HIV (PLHIV) is necessary and an important public health intervention especially in developing countries where both TB and HIV are serious problems and known to be synergistic and bidirectional. The World Health Organization (WHO) recommends the use of at least six months Isoniazid preventive therapy (IPT) for PLHIV without active TB in countries with a high burden of TB infection [1]. Ethiopia is classified as one of the world’s high TB burden, high multi-drug resistant-TB burden and high TB-HIV burden country by WHO [2]. IPT implementation in the country started in 2005 [3]. Per national guidelines, all PLHIV should be screened for active TB disease using the WHO four-symptom based screening (current cough, fever, night sweats or weight loss). If no evidence of active TB is found, IPT should be provided [1, 3].

Many studies demonstrated the benefit of IPT among PLHIV [3–11]. A Cochrane review of 12 randomized controlled trials revealed that IPT lowers the incidence of active TB compared to placebo (relative risk 0.68, 95 % confidence interval (CI) 0.54 to 0.85) [3]. Recent studies in Ethiopia reported a reduction of about 50 % in tuberculosis incidence in patients using IPT [5, 6]. Despite the confirmed efficacy of IPT and existing global recommendations and policy by the WHO since 2004, its implementation remains limited. Globally, the number of PLHIV who were treated with IPT reached 933 000 in 2014, an increase of about 60 % compared with 2013 [2, 12]. Nonetheless, 77 % of countries did not report provision of IPT as part of HIV care in 2014.

The implementation guideline for TB/HIV collaborative activities in Ethiopia states that IPT can be given to PLHIV after screening for the absence of four TB suggestive symptoms (current cough, fever, night sweats and weight loss) and any contraindications [3]. To those for whom active TB is excluded, a daily, self-administered dose of isoniazid is given for a period of six months at a dose of 5 mg/kg for children and 300 mg/day for adults. IPT can be initiated before or after the initiation of antiretroviral therapy. In Ethiopia, the number of PLHIV newly enrolled in care who initiated IPT in 2014 was 10,385 (excluding the missing data for 3 of Ethiopia’s 11 regions) [12]. A study by Cowan et al. [13] reported low IPT implementation in the Tigray region of Ethiopia.

Different reasons have been pointed out for the low uptake of IPT implementation globally. These are amongst others, related to health service providers’ knowledge and perception about IPT provision, inadequate application of the symptom screening tool (intensified TB case finding) to rule out active TB, inadequate patient adherence to IPT and fear of amplifying resistance to isoniazid through IPT [12–17]. In Ethiopia limited evidence is available regarding the status of and barriers to IPT implementation. As IPT coverage is known to be limited, it would be important to determine the actual level of IPT implementation and the reasons for its low uptake.

## Methods

This study was conducted in Tigray region located in northern Ethiopia. This study setting was selected to further investigate the reports by Cowan et al. [13] which indicated low uptake of IPT implementation in the region. As per the 2014 health status report, the region had 102 health facilities providing care to PLHIV [18]. In the region, IPT was initiated late in the year 2010. Eleven public hospitals, offering IPT for at least three or more years were included in this study. A further three newly established hospitals as well as all the health centers in the region were not included as these health facilities were not providing IPT yet as per unpublished reports obtained from the Tigray regional health bureau.

A mixed approach of quantitative and qualitative study designs was employed. The quantitative section aimed to determine IPT coverage of the region while in-depth interviews and focus group discussions were used to investigate barriers for health workers to implement IPT. The quantitative data were collected by using a structured data collection form and review of patient records. Information on availability of isoniazid, functionality of x-ray machine, stock out of reagents and slides required for the diagnosis of TB was collected from each hospital. Information on the number of PLHIV registered in HIV care and provided with IPT between September 2011 and April 2014 was collected from IPT registries of each hospital. IPT coverage was determined as percentage of PLHIV started on IPT among all PLHIV registered in HIV care.

Health providers (physicians, nurses, health officers, pharmacy workers and laboratory staff) were the interviewees and all participated in the focus group discussions. Health providers who were present during the time of the visit of the field team to the 11 hospitals and having at least 5 months working experience in HIV care were included in the qualitative study. The study participants were approached with the assistance of the trained study focal person in each hospital, selected and trained to collect data. The focal persons were recruited among nurses and pharmacists working in the 11 hospitals but not in the HIV clinic. The focal persons were trained as data collectors on how to approach study participants, how to ask for informed consent, conduct interviews and focus groups, collect quantitative data from patient records while maintaining the confidentiality of the collected data. The in-depth-interviews and focus groups were conducted in a separate room in each hospital close to the HIV clinic to ensure privacy. Each interviewee was first asked individually about his/her experience of providing IPT and challenges faced in its implementation. This was then followed by a focus group discussion to identify common barriers hindering implementation of IPT. Each in-depth interview took about 25 min while focus groups had an average duration of about 35 min till saturation was reached. The focus group discussions were moderated by the investigators. All the planned in-depth interviews in the 11 hospitals (three to five interviews in each hospital) were conducted. A total of 8 representative focus groups were conducted (one focus group per hospital) till saturation was reached. Data were collected between April and August, 2014.

Data from both quantitative and qualitative parts were analyzed by the investigators. The quantitative data were analyzed using SPSS for windows version 20 statistical software. Data was checked for completeness and accuracy through proofreading while entering the data into the software. Descriptive statistics (frequencies and percentage) were employed and data were visualized using tables and bar graphs. Audio-recorded data from the in–depth interviews and focus groups were transcribed using verbatim and translated into English. Qualitative data verification for accuracy and completeness was done through reading and re-reading by different investigators to ensure all recorded information and variations were identified. After transcription, codes were developed by two investigators based on the original terms used, and were matched. The transcripts and notes were analyzed thematically by categorizing them in line with the specific objectives (providers, patient, and resource requirement related barriers for implementation of IPT). The codes were presented, discussed and checked within the research team. Tentative categories and sub-categories were created from the clustered codes, and subsequently main themes emerged based on the patterns and relationships between the categories. Main themes were illustrated with representative quotations.

## Results

Fifty health providers participated in the qualitative study. The mean age (±SD) of health providers was 31 (±6) years. Twenty four (48 %) of the participants were nurses. Thirty (60 %) of health providers had more than two years working experience in HIV care (Table 1).

**Table 1:** Demographic characteristics of health providers interviewed at 11 HIV clinics of Tigray, Northern Ethiopia, 2014

Demographic characteristics		Health providers (*N = 50*)	
		N	%
Age (yrs)	<30	25	50.0
	30–60	25	50.0
Sex	Female	21	42.0
	Male	29	58.0
Profession	Nurse	24	48.0
	Health officer	7	14.0
	Medical doctor	4	8.0
	Pharmacy	10	20.0
	Laboratory	5	10.0
Work experience in HIV care (yrs)	<2	20	40.0
	≥2	30	60.0

All the 11 hospitals reported having a functional chest x-ray machine. Four hospitals (36.4 %) reported at least once a stock out in the past one year of microscopic reagents and/or slides for the diagnosis of TB. In the year 2013, shortage of adult and pediatric dose of isoniazid was reported in seven and four of the 11 hospitals respectively. This resulted in interruption of IPT among PLHIV already on IPT and limited initiation of IPT for new PLHIV.

**Fig. 1:**
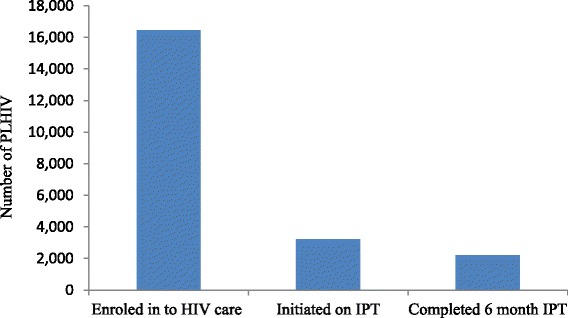
Number of PLHIV provided with IPT in 11 hospitals of Tigray, Northern Ethiopia, September 2011- April 2014

Out of the 16,443 PLHIV registered in the HIV clinics between September 2011 and April 2014, 3230 (19.6 %) had initiated IPT and 68 % of them had documentation on completion of six month IPT (Fig. 1). Despite the adoption of IPT policy in the country since 2005, health facilities included in this study started implementation in the years 2010 to 2012. IPT coverage varied between hospitals; two hospitals had high IPT coverage (of 47.0 % and 36.2 %) while two other hospitals had a much lower coverage (of 8.1 % and 9.2 %). Also, four of the 11 hospitals were not providing IPT at the time of the study due to isoniazid stock outs. The shortage had been experienced since about two months; some PLHIV already on IPT were forced to discontinue their treatment. Besides interrupting treatment for those on IPT, it limited initiation of IPT for new PLHIV. Low IPT coverage was more seen among the facilities that started IPT implementation late, in 2012 (Table 2).

**Table 2:** IPT start year and coverage of 11 hospitals of Tigray, Northern Ethiopia, September 2011-April 2014

Health facility	IPT start year	Number of PLHIV registered	Number of PLHIV on IPT at the time of study	Total number of PLHIV provided IPT 2011 to 2014	IPT coverage (%)^a^
Hospital 1	2012	1734	0	159	9.2
Hospital 2	2012	1386	75	201	14.5
Hospital 3	2012	1320	58	159	12.0
Hospital 4	2012	1094	19	515	47.0
Hospital 5	2010	3229	0	634	19.6
Hospital 6	2011	1460	43	529	36.2
Hospital 7	2012	1327	45	108	8.1
Hospital 8	2012	954	0	142	14.9
Hospital 9	2011	776	6	186	24.0
Hospital 10	2012	1843	60	188	10.2
Hospital 11	2011	1320	0	409	31.0
Total		16,443	306	3, 230	19.6

*PLHIV* people living with HIV, *IPT* isoniazid preventive therapy

^a^IPT coverage was calculated by dividing the number of PLHIV registered in a particular health facility to the total number of PLHIV provided IPT

### Challenges in IPT implementation

#### Irregularity of isoniazid supply and fear of drug resistance

Although most providers believed that IPT can be effective, multiple factors had impeded implementation of IPT in the region. Most concerns raised were about the unreliable provision of isoniazid causing fear of isoniazid resistance. This was raised as a concern by almost all health providers during all focus groups as illustrated by the following quotes:

*‘One time they (regional administrators) say go ahead, next time shortage happens after patients take for two or three months. Many patients are stopping it without completion. We can’t be sure of the continuity of supply that is why we usually hesitate to initiate new ones’ –*health officer

One participant emotionally said *‘I don’t know! Are we helping or hurting our patients? There is irregular supply of isoniazid. It was the first phase patients who completed 6 month treatment. The others had interruptions after taking two or three months. This may lead to isoniazid resistance’ –*doctor

*‘…despite the current availability of sufficient isoniazid, there are some reservations in initiating IPT for new patients due to fear of future stock outs’ –*pharmacist

*‘Since I started working in this hospital, I had* never *provided IPT. Either there is no medication or it’s insufficient to our clients. We have strong fear of resistance development. Stock out is a common problem in our hospital’ –*health officer

*‘…we start today the interruption follows tomorrow. I believe the risk of resistance may outweigh its benefit. Our patients are also losing trust in us. When we told them (the patients) the medication is not available, they feel as they might be exposed to danger’ –*nurse

*‘It is not easy to identify those patients who need IPT. There could be missed opportunities. Providing this single drug on top of the stock outs, obviously resistance may develop’ -*doctor

*‘We sent drug susceptibility test for patients with presumed multi-drug resistant TB and they become isoniazid resistant. I always question if the isoniazid we are providing for prophylaxis is contributing to such resistance. We hesitate in initiation. Unless it’s supported by data, it’s difficult to say IPT is being beneficial … –*health officer

*‘Previously there were problems of awareness in most of us (exposure of patients to a single drug isoniazid may lead to resistance). But currently there is no such fear as far as we are treating latent TB. And the problems of on-off supply are currently corrected’ –*pharmacist

#### Policy implementation gaps

There are gaps between having the IPT policy in place and the actual implementation. The policy for IPT among PLHIV implementation was adopted nationally and implementation started in Ethiopia since 2005; however, the participants report that the policy was only implemented in Tigray region since 2011 due to fear of amplifying resistance to isoniazid through IPT. Health authorities’ concern regarding isoniazid resistance in the region still exists due to irregularities in the drug supply. Not all HIV clinics in the region were permitted by the regional health authorities to offer IPT. At the time of this study, health centers providing antiretroviral therapy were not permitted to offer IPT, IPT was only being offered in hospitals. Lack of commitment of the health authorities and drug supply irregularities were raised by the service providers as reasons for the gap between having a policy and actual implementation. Health service providers also reported poor monitoring and lack of supervision of the IPT program by higher managers.

The following quotes illustrate the above interpretation.

*‘IPT is a sensitive issue in this region because one time the region was refusing its use and was criticized as the only region not implementing. One time I remember the regional health authorities refusing its use unless there is continuous supply from pharmaceutical fund and supply agency (PFSA) …’ -*nurse

*‘We ask isoniazid every two months from PFSA. But most of the time they had stock outs. I think there is a problem in the system. Priority should be given to PLHIV…’ –*pharmacist

*‘…IPT is not being considered as prime treatment for PLHIV as antiretroviral therapy and cotrimoxazole. This is mainly the problem of the government and suppliers. If we can convince our patients that IPT will help them and the government is committed to supply isoniazid, it is possible to prevent tuberculosis in our patients’ –*pharmacist

*‘Previously IPT was not being provided to our patients. Now we have started it. There are problems of commitment from responsible bodies. We ourselves didn’t push others as such for its implementation. I believe we don’t have a problem of supply, it’s due to lack of giving attention for the program. There are problems of convincing its’ importance to our patients. There are also problems of following the implementation by higher authorities. IPT should be considered as prime issue’ –*health officer

#### Drug side effect, pill burden and adherence related barriers

Providers reported that the unavailability of the adult dose of isoniazid resulted in a pill burden for PLHIV as instead of one, three tablets of the pediatric doses were provided to adults. This was compromising overall adherence. Most providers indicated that they prescribed IPT based on the patients’ willingness and they indicated patients’ were unwilling to add isoniazid to the antiretroviral therapy due to fear of having many pills.

*‘Sometimes it’s only the 100 mg isoniazid lefts in our stock. Providing three tablets for an adult is resulting in pill burden. Patients complain having many medications, and some* gastrointestinal *irritations. Hence, some patients are not willing to take it’ –*pharmacist

*‘We had two patients who developed TB while on IPT. Such cases could be either a missed one during screening or due to immune reactivation in a patient with a low CD4 count or it could be because of patient non-adherence…’ –*doctor

Side effects such as gastrointestinal irritation, peripheral neuropathy and hepatotoxicity were also mentioned as challenges in IPT implementation. Some of the side effects are preventable however unavailability of pyridoxine had contributed to the problem of neuropathy.

*‘…there is also shortage of pyridoxine. This could lead to increased risk of peripheral neuropathy…’ –*pharmacist

*‘We had patients who interrupted the treatment due to shortage. Even currently we are not providing it. Its implementation is poor. It’s only initiation, we never see a patient completing it. There is a big problem in supply. There are also some patients who interrupted IPT due to side effects such as hepatotoxicity and peripheral neuropathy’ –*nurse

#### Lack of training and guidelines

Health providers also reported the absence of training opportunities as well as availability of guidelines with regards to IPT. They believed that training should be provided first before the introduction of a program and it should be continued thereafter after for new staffs.

*‘We did not take special training regarding IPT. We have been trained within the antiretroviral therapy package and we don’t have special guideline…’ –*nurse

*‘Training and update on IPT are lacking. I had never seen a new guideline, which are important to make us more confident’ –*health officer

*‘Also we are not trained in this regard. It was by letter they (regional health authorities) told us to start IPT’ –*nurse

**Fig. 2:**
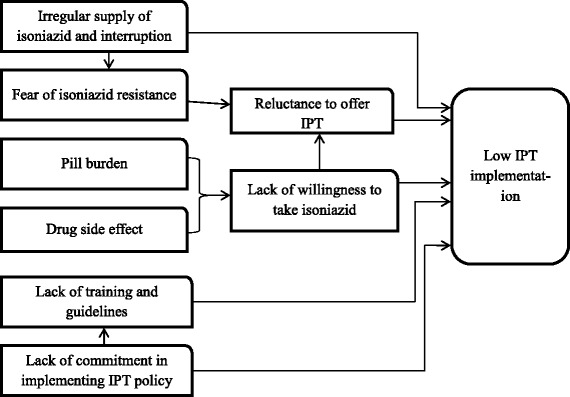
Conceptual framework of barriers to implementation of IPT among PLHIV in Tigray, Northern Ethiopia, 2011–2014

The main barriers to IPT implementation underpinning the current research are summarized in a conceptual framework (Fig. 2).

## Discussion

Implementation of IPT in the Tigray region of Ethiopia was started late and coverage was limited despite the fact that the policy has been in place in the country since 2005 [3]. The barriers hindering implementation of the IPT program were predominantly related to isoniazid supply problems, patient acceptance, concerns about amplifying resistance to isoniazid through IPT, and lack of commitment of health managers to scale up its implementation.

In this study only about one fifth (20 %) of PLHIV registered in care were provided with IPT. This is lower than the reported IPT coverage of around 30 % for Addis Ababa in 2011 [19, 20]. Differences in access to isoniazid could have contributed to the lower coverage observed in the northern region of Tigray as supply could have been better in Addis Ababa, the capital city of Ethiopia. Another reason for this difference could be related to the denominator used. In our study, IPT coverage was determined with all PLHIV registered in the HIV clinics as denominator, whereas the Addis Ababa studies calculated the coverage with the eligible as denominator this excluding those illegible to receive IPT. Our study was based on retrospective data, and eligibility of IPT could not be determined from the available routine records for all PLHIV therefore we adopted the wider denominator. Despite this difference, both results still indicate sub optimal coverage as the WHO target level of 50 % coverage [12] is not yet achieved.

The main barrier leading to sub-optimal implementation of IPT observed in this study was shortage of isoniazid. It seems that lack of effective systems to manage drug supply is impeding implementation of the policy. Shortage of essential drugs, such as drugs used for TB and HIV treatment and prevention has been reported as a problem in health facilities of Ethiopia [21–24]. Non-availability of isoniazid was also observed by Getahun et al. [12] as a barrier to global IPT implementation. The supply problem of isoniazid could arise at the level of quantification, procurement, and/or distribution. Participants in this study complained that they do not receive the quantities they requested. A study from Addis Ababa reported that only 47 % of the health facilities received the full quantity of drugs they ordered [24].

The success of IPT implementation is mainly dependent on continuous resource mobilization, capacity building and monitoring. Health providers perceived that insufficient attention is being given to IPT by responsible bodies. Most of the hospitals in this study started implementing the IPT program just because they were told do so by higher officials. In some hospitals sensitization workshops, technical trainings, and operational guidelines were not provided. Health care staff also felt that there was no adequate monitoring and evaluation of the policy implementation. Similarly a study in Addis Ababa [16] reported that lack of reinforcement by health officials and stakeholders working in TB was affecting implementation of the IPT policy. A study in South Africa also reported lack of awareness on the benefits of IPT and the guidelines and recommendations by prescribers as primary barrier to IPT implementation [17]. Provision of a clear up-to-date policy and provision of trainings has been found to improve IPT implementation in other studies [25–27]. Trainings and good communication are vital for proper understanding of the policy and avoid ambiguities on IPT provision among health providers. This study did not include regional health managers, therefore their perspective is missing.

Lack of confidence in excluding active TB and fear of resistance development has been a matter of debate for many years [28, 29]. The WHO strongly argues that concerns regarding the development of isoniazid resistance should not be a barrier for providing IPT [1] as the evidence disproves the development of isoniazid-resistant TB after providing IPT to PLHIV [28, 30]. However, fear of isoniazid resistance is still a challenge to IPT implementation. In settings with limited diagnostic capacity, concerns about resistance may arise when use of chest X-ray is considered important to exclude active TB disease [31]. Concerns regarding amplifying resistance to isoniazid through IPT have been pointed out as challenge in several other studies [12, 17, 26, 32, 33].

Low completion rates of IPT were observed in this study. The main reasons could be related to shortage of isoniazid, poor patient adherence and side effects. Unavailability of adult isoniazid dose resulted in pill burden when providers instead issued three tablets of the pediatric doses to make up the adult dose. This resulted in unwillingness of patients to take IPT due to a concern of having too many tablets. And in those willing to take it, patient adherence was questionable due to the pill burden. A comparable study in Addis Ababa [16] also observed that poor patient adherence was a major barrier affecting the implementation of IPT. Concerns regarding pill burden and inadequate patient adherence were also raised in other studies [12, 15, 34]. In this study peripheral neuropathy and hepatotoxicity were reported as potential side effects related to IPT resulting in some patients discontinuing the treatment. Providers perceived that lack of pyridoxine in the hospitals increased the prevalence of peripheral neuropathy. A study by Durovni et al. [27] reported that 1.2 % of persons initiating IPT discontinued therapy due to adverse reactions. However, the rate of hepatotoxicity associated with isoniazid was reported as low (0.001–0.15 %) in several studies [9, 12, 33, 35].

The current study provides insight into the main reasons underlying health providers’ reluctance to offer IPT like fear of resistance development due to frequent interruption of IPT, lack of clients’ willingness to take isoniazid, and uncertainties on the future supply of the drug. Concerns about resistance development through IPT had been disproved in previous studies. However, these studies were done with full supply of IPT and without any interruption. In the current study the concern of frequent interruption of IPT supply was a big challenge for the IPT program. Future studies should focus on the risk of isoniazid resistance development through IPT in settings with irregular supplies and frequent interruption of IPT. Finally, this study determined the actual coverage of IPT but also gained insight in teasing out the underlying factors, some of them have been observed in other studies. Key factor here is the drug supply issue which is fixable and concrete actions can be taken.

## Conclusions

Implementation of IPT in the Tigray region of Ethiopia was low with only one fifth of PLHIV covered. The main barrier mentioned hindering IPT implementation was irregular supply of isoniazid resulting in stock outs and interruption of IPT provision, creating fear among health care workers for development of drug resistance. Health managers, drug suppliers and partners working in HIV-TB programs should be committed to ensure an uninterrupted isoniazid supply for the success of IPT implementation.

## Additional file

Additional file 1: Dataset. (XLSX 26 kb)

## Abbreviations

CD4: Cluster differentiation
CI: Confidence interval
COREQ: Consolidated criteria for reporting qualitative research
ERC: Ethics Review Committee
HIV: Human immunodeficiency virus
IPT: Isoniazid preventive therapy
N: Number
PFSA: Pharmaceuticals Fund and Supply Agency
PLHIV: People living with HIV
SD: Standard deviation
TB: Tuberculosis
TRAC/FMOH: Tuberculosis Research Advisory Committee/Federal Ministry of Health
WHO: World Health Organization
